# Topical Anesthetics and Premature Ejaculation: A Systematic Review and Meta-Analysis

**DOI:** 10.7759/cureus.42913

**Published:** 2023-08-03

**Authors:** M Danial Ali Shah, Safdar Shah, Nadeem Bin Nusrat, Nauman Zafar, Assad Ur Rehman

**Affiliations:** 1 Department of Urology, Pakistan Kidney and Liver Institute and Research Center, Lahore, PAK; 2 Department of Urology, Evercare Hospital Lahore, Lahore, PAK

**Keywords:** systematic review, sexual health, topical anesthetics, premature ejaculation, meta-analysis

## Abstract

This meta-analysis was conducted to assess the effectiveness of topical anesthetics in preventing premature ejaculation. We conducted an online database search for original studies comparing topical anesthetic agents with placebo in patients with premature ejaculation. After selecting relevant articles, we extracted data on baseline characteristics and predetermined endpoints. Intravaginal ejaculatory latency time (IELT) was the primary outcome for efficacy. Mean differences and corresponding 95% confidence intervals were used to present continuous data. A random-effects model was used to pool the data, and subgroup analysis was performed based on the type of anesthetic agent used. Eleven randomized controlled trials were examined, involving a total of 2008 participants. After analyzing the combined results, it was found that Severance Secret (SS) cream (CJ CheilJedang Corporation, Seoul, South Korea) demonstrated significantly higher effectiveness than a placebo in increasing IELT (P = 0.001). Similarly, the topical eutectic mixture for premature ejaculation (TEMPE), lidocaine, and the eutectic mixture of local anesthetics (EMLA) were significantly more efficient than a placebo (P<0.00001; P = 0.0001; P<0.00001). Additionally, it was found that lidocaine gel was more efficient than paroxetine or sildenafil (P = 0.04; P<0.00001). In conclusion, topical anesthetics increase IELT in men with premature ejaculation more effectively than placebo, sildenafil, tadalafil, paroxetine, and dapoxetine.

## Introduction and background

The most prevalent male sexual dysfunction is premature ejaculation (PE), which impacts approximately 20-30% of males globally [1]. PE can lead to mental anguish, anxiety, shame, and depression, harming one's self-esteem and potentially straining their relationship with their partner [2,3].

PE had no previously established definition until it was formulated by the International Society for Sexual Medicine (ISSM) [1,4]. According to their description, PE is a sexual dysfunction in males marked by ejaculation that regularly happens either before or roughly within 60 seconds of vaginal penetration, an inability to postpone ejaculation in almost all or all vaginal penetrations, and unfavorable personal outcomes such as stress, dissatisfaction, and avoidance of sexual closeness [5].

PE is typically categorized as either a primary condition, which emerges at the start of sexual maturity and persists throughout life, or as a secondary condition, which arises after a period of normal sexual function [6]. The intravaginal ejaculation latency time (IELT) is regarded as the most reliable measure of improvement in PE, according to the most recent definition, which specifies that IELT should be less than one minute. As a primary endpoint frequently employed in studies on PE, IELT is a significant criterion.

There is a range of treatment options for PE, which may include behavioral and pharmacological interventions. Local anesthetics are one such treatment, which decreases the sensitivity of the glans penis and prolongs the time to ejaculation without negatively impacting the experience of ejaculation [7].

In researching the use of topical anesthetics in treating PE, it's essential to consider the availability of sufficient clinical evidence. To that end, we conducted a systematic evaluation and meta-analysis of existing data.

## Review

Methodology

The meta-analysis adhered to the Preferred Reporting Items for Systematic Reviews and Meta-Analyses (PRISMA) guidelines for systematic review and meta-analysis [8]. A thorough electronic search was conducted on PubMed, Scopus, and Cochrane CENTRAL up to June 3, 2023, with no limitations on time and language. The investigation was performed using keywords such as “premature ejaculation,” “early ejaculation,” “rapid ejaculation,” “anesthetics,” “local anesthetics,” and “topical anesthetics.” To find any additional pertinent studies, we manually searched the reference lists of the trials we found and earlier meta-analyses and review articles.

Study Selection

Only randomized controlled trials (RCTs) that satisfied the condition of comparing the effectiveness and safety of topical anesthetic agents in patients who had PE were considered. However, RCTs that involved patients with PE caused by organic factors such as thyroid disease, erectile dysfunction (ED), anatomical abnormality, substance or alcohol abuse, hypertension, diabetes mellitus, renal failure, psychiatric illness, and genital infection were excluded.

Data Extraction and Assessment of Study Quality

After conducting a systematic search, the articles were transferred to the EndNote Reference Library software (Clarivate Plc. London, United Kingdom). Any duplicate reports were identified and removed. Two reviewers carefully evaluated the remaining articles and only included trials that fulfilled the pre-defined criteria. The trials were initially screened based on their title and abstract, and those considered relevant were reviewed in full. In case of any discrepancies, a third investigator was consulted to resolve them. To evaluate the quality of the trials published, the modified version of the Cochrane Collaboration's risk of bias tool for randomized controlled trials was utilized [9].

Statistical Analysis

RevMan version 5.4 (2020; The Cochrane Collaboration, The Nordic Cochrane Centre, Copenhagen, Denmark) was utilized for all statistical analyses. The mean difference with 95% confidence intervals (CIs) was used to present the trial results, and a random effects model was used to pool them. The method outlined by Hozo et al. [10] was used to calculate the standard deviations or standard errors if they weren't reported in the trial.

Forest plots were generated to evaluate the pooling results visually. Any differences between the subgroups were found using the chi-square test. Higgins I2 was used to measure study heterogeneity, and a value of less than 50% was considered acceptable. To assess publication bias, Begg's test and a visual inspection of the funnel plot were utilized. A significance level of less than 0.05 was considered to account for each case.

Results

Literature Search Results

**Figure 1:**
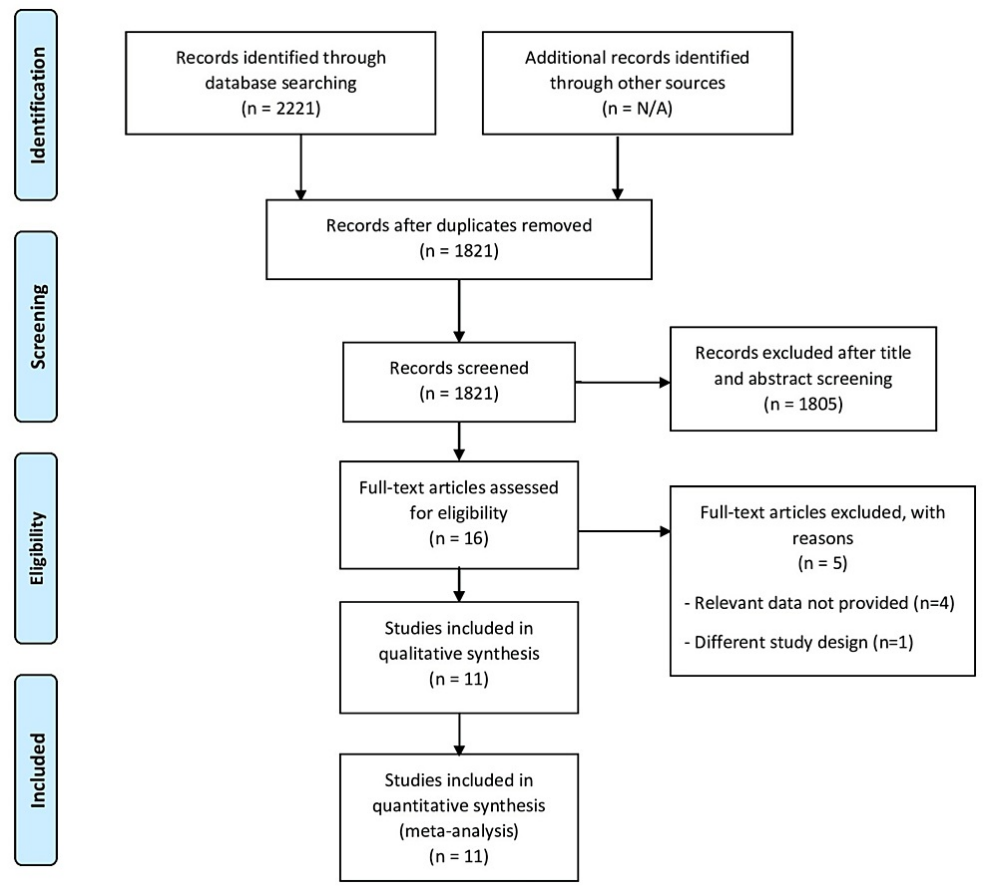
PRISMA flowchart of selection of studies. PRISMA, Preferred Reporting Items for Systematic Reviews and Meta-Analyses

An initial search of three electronic databases resulted in 2221 potential studies. Of these, 16 articles were obtained in full text as potentially relevant. The PRISMA flow diagram shown in Figure 1 describes the complete process of selecting studies. The analysis identified 11 RCTs that examined the effectiveness of a topical anesthetic agent compared to a placebo or another drug. The details of the RCTs included in the analysis are provided in Table 1.

**Table 1:** Characteristics of included studies. TEMPE: topical eutectic mixture for premature ejaculation; IELT: intravaginal ejaculatory latency time; NR: not reported; EMLA: eutectic mixture of local anesthetics; ISSM: international society of sexual medicine; IEC: index of ejaculatory control; PEP: premature ejaculation profile; RCT: randomized controlled trial; PC: pre-coitus; DSM: the diagnostic and statistical manual of mental disorders; SHIM: sexual health inventory for men; SQol: sexual quality of life; AIPE: Arabic index premature ejaculation; SD: standard deviation; SS: Severance Secret*; SSR: sexual satisfaction ratio; ED: erectile dysfunction; IPE: index of premature ejaculation *CJ CheilJedang Corporation, Seoul, South Korea

Author	Year	Country	Duration	Inclusion criteria	Exclusion criteria	Mean age (Yr.) ± (SD)	Intervention, control, numbers	Efficacy outcomes	Adverse events
Choi et al. [11]	1999	Korea	6 months	IELT <3min, SSR<50% in both patients and partners, lifelong PE only	Acquired PE, ED, Infections, neurological, psychiatric disorders, and drugs	37.1 ± 1	SS-cream (0.05g) 60 min PC, n=50	IELT, SSR	7
							SS-cream (0.10g) 60 min PC, n=50		9
							SS-cream (0.15g) 60 min PC, n=50		7
							SS-cream (0.20g) 60 min PC, n=50		10
							Placebo, n=50		4
Choi et al. [12]	2000	Korea	NR	IELT <3min, SSR<30% in both patients and partners, lifelong PE only	Acute/Chronic illness, any intervention/medication for treating PE in the last two months	38.7 ± 0.61	SS-cream (0.2g) 60 min PC, n=106	IELT, SSR	110
							Placebo, n=106		0
Atikeler et al. [13]	2002	Turkey	NR	IELT <1 min, Lifelong PE	Infections, DM, HTN, psychiatric disorders, and drugs	29.4 ± (NR)	EMLA (2.5g) 20 min PC, n=10	IELT	0
							EMLA (2.5g) 30 min PC, n=10		6
							EMLA (2.5g) 45 min PC, n=10		10
							Placebo, n=10		0
Busato et al. [14]	2004	Brazil	4-8 weeks	DSM-IV, lifelong and acquired PE	PE due to organic cause, ED	33.4 ± (NR)	EMLA (2.5 g), 10-20 min PC, n=16	IELT, SSR	5
							Placebo, n=13		0
Dinsmore et al. [15]	2007	UK and Netherlands	4 weeks	DSM-IV, Lifelong PE,	ED	38.5 ± 9.8	TEMPE: lidocaine (7.5 mg) and prilocaine (2.5 mg) 15 min PC, n=20	IELT, IEC, and SQoL change	6
							Placebo, n=23		4
Dinsmore et al. [16]	2009	Europe	12 weeks	ISSM and DSM-IV, acquired and lifelong PE	ED, use of psychiatric medications, G6PD	35 ± 9.6	TEMPE (7.5mg lidocaine, 2.5mg prilocaine) 5min PC, n=191	IELT, PEP questionnaire	18
							Placebo, n= 99		3
Carson et al. [17]	2010	Canada, Poland, USA	12 weeks	ISSM and DSM-IV, acquired and lifelong PE	ED	39.1 ± 11.7	TEMPE (7.5mg lidocaine, 2.5mg prilocaine) 5min PC, n=167	IELT, IPE, PEP	17
							Placebo, n=82		1
Gameel et al. [18]	2013	Egypt	4 weeks	IELT of <2min, PE > 1 year	ED, On medications for psychiatric problems,	32.8 ± 3.8	Lidocaine gel (15 min PC) + oral vitamin (1-4h PC), n=30	IELT, Sexual Satisfaction score	22
							Tramadol 50mg (2h PC) + inert gel (15 min PC), n=29		0
							Sildenafil 50mg (1 h PC) + inert gel (15 min PC), n =30		0
							Paroxetine 20mg (4 h PC) + inert gel (15 min PC), n=28		0
							Placebo (oral vitamin 1-4 h PC) + inert gel (15 min PC), n=27		0
Dell'atti et al. [19]	2017	Italy	3 months	ISSM definition, Lifelong PE	ED, use of psychiatric medications, metabolic disorders	32.4 ±9.5	Lidocaine Spray (10g/100ml) 5 min PC, n=25	IELT, Sexual Satisfaction score	4
							Tadalafil 5mg OD, n=27		5
							Tadalafil 5mg OD + lidocaine spray 5min PC, n=26		7
El-Hamd et al. [20]	2020	Egypt	8 weeks	ISSM definition, Lifelong PE	ED, Neurological or psychiatric disorders, DM	33.88 ± 5.42	Lidocaine spray 5% (one-two actuation) 10-20 min PC, n=75	IELT, AIPE	6
							Placebo, n=75		0
Alghobary et al. [21]	2020	Egypt	12 weeks	Lifelong PE, IELT < 1 min	ED, chronic medical and psychological disorder	37.7 ± 6.2	Lidocaine 10% (3-5 puffs) 10 mins PC, n= 28	IELT, AIPE, and SHIM scores	38
							Dapoxetine 60mg 1-3 hr PC, n=27		35

Study Characteristics and Quality Assessment

**Figure 2:**
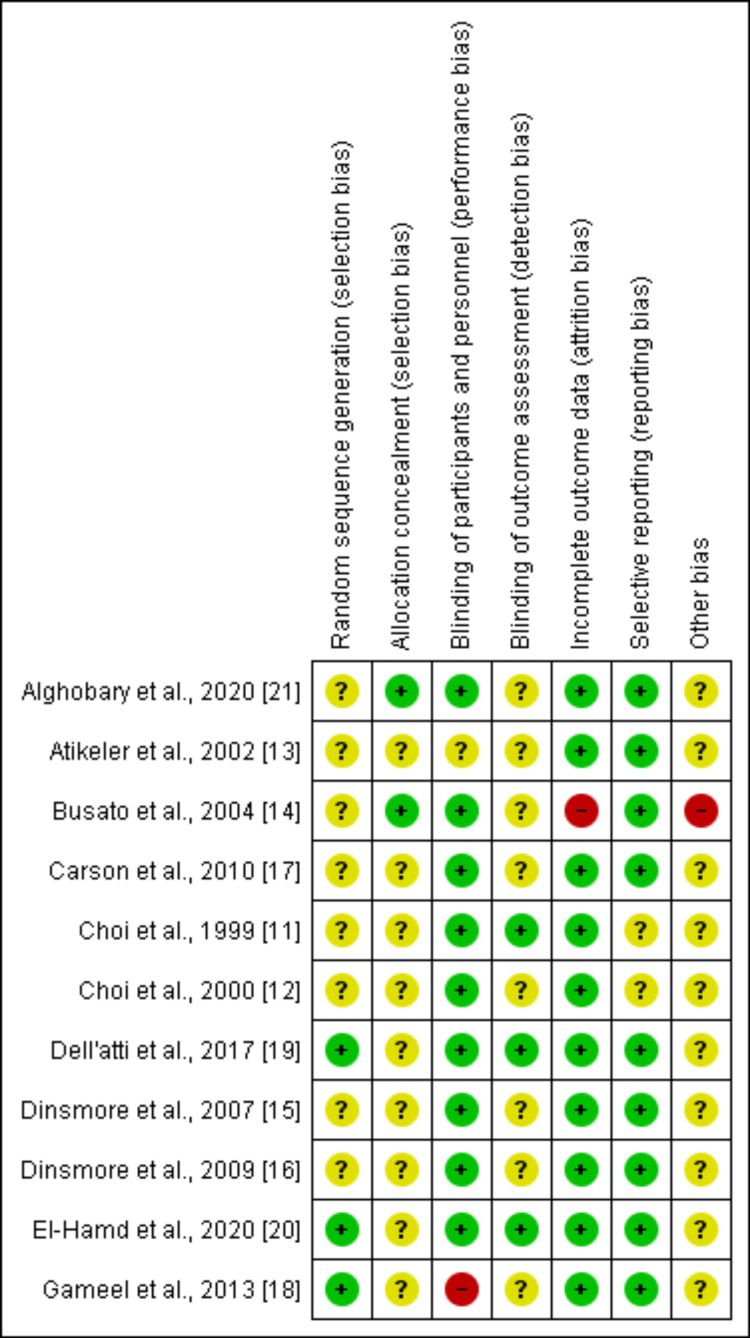
Risk of bias assessment summary. References: [11-21]

The quantitative analysis involved 11 studies with a total of 2008 participants. IELT was used as a measure of PE. The mean age of the individuals in the pooled sample was 35, with a standard deviation of 6.4 years. The risk of bias assessment is shown in Figure 2.

Comparison of Topical Anesthetics with Placebo

**Figure 3:**
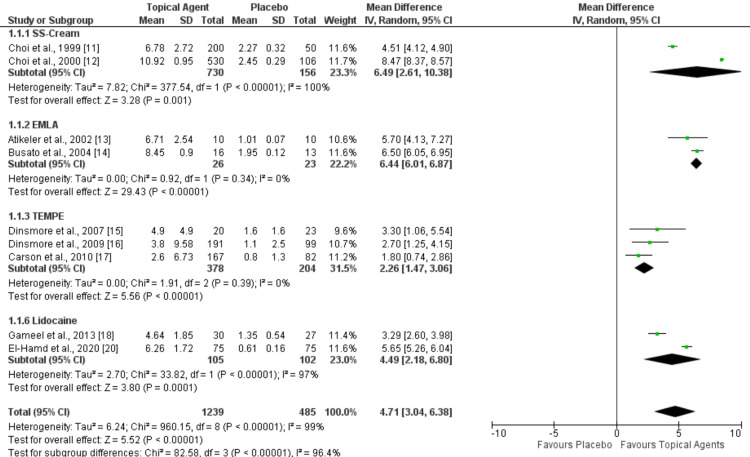
Forest plot comparing the effect of topical agents and placebo on IELT. SS: Severance Secret*; EMLA: eutectic mixture of local anesthetics; TEMPE: topical eutectic-like mixture for premature ejaculation; IELT: intravaginal ejaculation latency time References: [11-18, 20] *CJ CheilJedang Corporation, Seoul, South Korea

A meta-analysis of two RCTs (n = 886) examined the effect of applying SS cream 60 minutes before intercourse on the mean IELT. The pooled mean difference in IELT was 6.49, significantly favoring SS cream (95%CI 2.61-10.38; P < 0.00001). Low heterogeneity (I2=0%) was seen in the meta-analysis of two RCTs (n=49) for the eutectic mixture of local anesthetics (EMLA) cream subgroup. In favor of EMLA, the pooled mean difference in IELT was 6.44 (95%CI 6.01-6.87; P<0.00001). In the topical eutectic mixture for premature ejaculation (TEMPE) subgroup, the meta-analysis of three RCTs (n=582) showed low heterogeneity (I2=0%). The pooled mean difference in IELT was 2.26 (95%CI 1.47-3.06; P<0.00001). In the lidocaine subgroup, the meta-analysis of two RCTs (n=207) showed a pooled mean difference in IELT was 4.49 (95%CI 2.18-6.80; P<0.0001), which favors lidocaine over placebo. Figure 3 shows the forest plot for these analyses.

Comparison of Topical Anesthetics with Oral Agents

**Figure 4:**
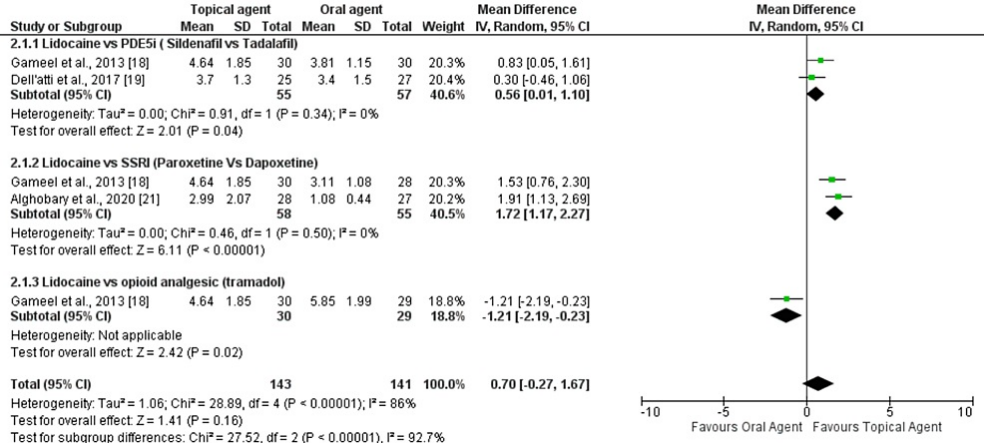
Forest plot comparing the effect of topical agents and oral agents on IELT. PDE5i: phosphodiesterase type 5 inhibitor; SSRI: selective serotonin reuptake inhibitor; IELT: intravaginal ejaculation latency time References: [18-19, 21]

The meta-analysis result showed lidocaine to be more effective than oral agents except when compared with tramadol. The mean difference in IELT favored lidocaine gel over sildenafil with a value of 0.56 minutes (95%CI 0.01-1.10; P<0.04), and lidocaine gel exhibited a more significant advantage over paroxetine and dapoxetine with a mean difference of 1.72 minutes (95%CI 1.17-2.27; P<0.00001). Tramadol demonstrated significantly greater effectiveness than lidocaine gel, with a mean difference of 1.21 minutes (95%CI 2.19-0.23; P=0.02). Figure 4 shows the forest plot for these analyses.

Discussion

Our analysis of 2008 participants provides compelling evidence of local anesthetics’ efficacy for PE. It showed that topical anesthetics are better than placebo and oral agents in preventing PE except when compared with tramadol. In this meta-analysis of 11 clinical trials, all studies were randomized and prospective, using a stopwatch to measure IELT.

In the past, the origins of PE were classified into two categories: psychogenic factors and biogenic factors, with psychogenic factors being regarded as the primary cause. According to a suggestion by Xin et al., the development of PE was associated with increased sensitivity of the penis, as demonstrated in studies using penis biothesiometry [22]. This finding served as the foundation for utilizing topical anesthetic agents to address PE. Consequently, treating primary PE could involve reducing the sensation of the penis through desensitization methods.

In our review, we identified four types of local anesthetic agents used for delaying ejaculation. These include SS-cream (a mixture of *Bufonis veneum* extract, *Asiasari radix* extract, *Caryophylli flos* extract, *Cinnamomi cortex* extract, and Ginseng Radix Alba fructose), TEMPE or PSD502 (a combination of 7.5 mg prilocaine eutectic mixture and 22.5 mg lidocaine), EMLA (a cream consisting of 2.5 g prilocaine eutectic and 2.5 g lidocaine), and lidocaine spray 5%. These drugs work by either desensitizing the penis or delaying the ejaculation reflex.

Topical anesthetic agents were found to have specific side effects that were not commonly observed with alternative treatments. These unique side effects included reduced sensation in the penile shaft, localized irritations, numbing of the partner's vagina, and loss of erection. Our review supports the undeniable effectiveness of topical anesthetics for treating PE, which is consistent with other reputable studies [23-25].

Study Limitations

There are some limitations to our meta-analysis. While sub-group analyses were conducted to mitigate or elucidate the substantial heterogeneity observed, this study's primary limitation remains the trials' heterogeneity. Furthermore, the definitions of PE used in the studies included in our analysis exhibited significant variability, which could pose another limitation and impact the interpretation of heterogeneity among the sources. Lastly, the varying sample sizes in the included studies resulted in different levels of statistical power.

## Conclusions

Given the substantial impact of premature ejaculation on quality of life and intimate relationships, it becomes crucial to explore and embrace effective treatment options. Topical anesthetics emerge as a game-changer in the field, providing tangible benefits beyond placebo effects. These anesthetic agents show efficacy and tolerability in PE patients, resulting in substantial improvements in IELT compared to a placebo. So it can be a viable treatment option in patients with PE.
